# Hepatitis A virus infection in Brazilian correctional facilities

**DOI:** 10.1371/journal.pone.0283868

**Published:** 2023-04-25

**Authors:** Lisie Souza Castro, Grazielli Rocha de Rezende, Marco Antonio Moreira Puga, Larissa Melo Bandeira, Tayana Serpa Ortiz Tanaka, Sabrina Weis-Torres, Deborah Ledesma Taira, Luiz Henrique Ferraz Demarchi, Julio Rosa Henrique Croda, João Renato Rebello Pinho, Michele Soares Gomes-Gouvêa, Ana Rita Coimbra Motta-Castro

**Affiliations:** 1 Universidade Federal de Mato Grosso do Sul, Campo Grande, Mato Grosso do Sul, Brazil; 2 Universidade Federal de Rondonópolis, Rondonópolis, Mato Grosso, Brazil; 3 Laboratório Central de Saúde Pública de Mato Grosso do Sul/SES/MS, Campo Grande, Mato Grosso do Sul, Brazil; 4 Fiocruz Mato Grosso do Sul, Fundação Oswaldo Cruz, Campo Grande, Mato Grosso do Sul, Brazil; 5 LIM-07, Hospital das Clínicas, Faculdade de Medicina, Universidade de São Paulo, São Paulo, São Paulo, Brazil; CEA, FRANCE

## Abstract

Hepatitis A virus (HAV) infection is transmitted by the fecal-oral route, through interpersonal contact and ingestion of contaminated food or water. Prisoners are at higher risk of acquiring HAV infection mainly due to the environment of closed penal institutions and socioeconomic conditions. This study aims to determine the seroprevalence of anti-HAV and its associated risk factors among inmates from twelve prisons in Central Brazil. A cross-sectional study was conducted between March 2013 and March 2014. A total of 580 prisoners participated in the study. The participant’s samples were tested for Total and IgM anti-HAV antibodies by electrochemiluminescence immunoassay (ECLIA). Risk factors associated with anti-HAV seropositivity were also analyzed. The prevalence rate of HAV exposure was 88.1% (95% CI: 85.5–90.7). No sample had a positive reaction to IgM anti-HAV. Increasing age, low level of education, and being imprisoned in Corumbá city were independently associated with HAV exposure among prisoners. To prevent the burden of the disease, vaccination strategies should be considered for susceptible prisoners in Central Brazil.

## Introduction

Hepatitis A virus (HAV) infection is the most common cause of acute hepatitis worldwide. This infection is transmitted through the fecal-oral route, including ingestion of food or water contaminated by this virus and interpersonal contact [1]. Sexual behaviors (such as oro-anal contact and a high number of sexual partners) have been reported as an essential factor that leads to HAV spread and rises the risk of acquiring HAV infection [2, 3]. High prevalence and incidence rates are also related to socioeconomic factors influenced by poor access to potable water and sanitation. While during childhood, most of the cases are asymptomatic, the HAV infection later in life is associated with higher severity of the clinical disease. Besides, preexisting chronic liver disease is also an important risk factor for severe/fulminant HAV infection [1, 3].

The Brazilian HAV endemicity has changed to low and intermediate depending on the region of the country. This epidemiological setting increases the susceptibility to HAV in adults, as well as the burden of the disease [1]. An immunization program against HAV was introduced by the Ministry of Health of Brazil only in 2014. Currently, a single-dose vaccine is offered to children between one and five years old [4]. People considered to be at high risk of acquiring and transmitting HAV infection are mainly men who have sex with men (MSM), sex workers, people who inject drugs (PWID), incarcerated persons, and travelers from endemic areas [5].

The prison population is considered a marginalized group, mainly due to low socioeconomic status and adverse sanitary conditions, before and during incarceration [6, 7]. Brazil has the fourth-largest prison population in the world, with approximately 700,000 inmates and an exceeded prison occupancy level of 165.1% [8]. Thus, with the overcrowding setting of Brazilian prisons and the high mobility of people, this environment becomes a reservoir of infectious diseases [6, 7]. Moreover, the Brazilian vaccination program against hepatitis A does not cover these groups. To determine the HAV exposure impact among prisoners from twelve closed penal institutions in Central Brazil, the study aimed to investigate the prevalence of anti-HAV antibodies and the main associated risk factors.

## Material and methods

The study population consisted of inmates who participated in a previous cross-sectional survey on HCV infection conducted among individuals in closed penal institutions of Mato Grosso do Sul State, Central Brazil [9]. Of the 21 penitentiaries in the State, 12 were included in the study and are in five different cities as follows: Campo Grande (5), Corumbá (2), Ponta Porã (2), Dourados (1), and Três Lagoas (2). A total of 3,368 inmates were enrolled in the survey between March 2013 and March 2014, representing 46.6% of prisoners from the twelve mentioned penitentiaries during the study period. All participants who read and signed the informed consent form were interviewed via a standard questionnaire containing information about variables of interest. Blood samples were collected from all individuals and the serum was used to perform serological tests [9].

Thus, according to the sample size calculation based on the estimated prevalence for total anti-HAV of 86% [6] (95% significance level; 3% accuracy; the power of 80%), the present study should include at least 480 prisoners. Retrospectively, a random selection of 580 participants from a total of 3,368 inmates was performed by individuals stratified proportionally by each prison using Epi-Info 6.04 software (CDC, Atlanta, GA, USA).

HAV exposure (current or exposure) was defined as a positive total anti-HAV and/or anti-HAV IgM test result. Participants lacking total anti-HAV and IgM anti-HAV were considered serologically susceptible to HAV infection. The samples were tested for total and IgM anti-HAV by electrochemiluminescence immunoassay (ECLIA) method on Roche Cobas® e 411 analyzers. Screening by enzyme-linked immunosorbent assay was also performed for anti-*Treponema pallidum* (ELISA- ICE Syphilis, DiaSorin, Italy), total anti-HBc, serological evidence of HBV infection (anti-HBc PLUS Bio-Rad, France), anti-HCV marker (ELISA—Murex Diagnostics, UK), and HIV-1/2 infection (Murex HIV-1.2.0, DiaSorin, Italy). The Federal University of Mato Grosso do Sul Ethics Committee on Human Research approved this study under protocol number 1.250.214 –CAAE: 49368115.9.0000.0021.

The variables evaluated were analyzed using STATA statistical software, version 13 (Stata Corporation, College Station, TX, USA). Continuous variables were expressed as median and range. Categorical data were analyzed using the Chi-square test or Fisher’s exact test. Odds ratios and 95% CI were used to measure the strength of the association between anti-HAV positivity (outcome) and the independent variables. Variables with a p-value of 0.20 or less were included in the logistic regression backward stepwise model, and Statistical significance was assessed at a p-value <0.05.

## Results

A total of 580 incarcerated persons were included in the study population. The median age of participants was 29 years (range 18–75; standard deviation [SD] ± 9.27), including 487 males (84.0%) and 93 females (16%). Most inmates reported tattoos (68.9%), having a stable partner (56.2%), and having no more than nine years of schooling (69.8%). Before the incarceration, the median number of individuals living in the same household with the study individuals was 4 (range 0–15; SD ± 2.22). About half of the participants (49.8%) declared illicit drug use, and 55.0% consumption of alcohol. Injection drug use and a history of blood transfusion were reported by 0.2% and 14.3% of participants, respectively. Regarding imprisonments, most of the participants (58.8%) declared having already been imprisoned previously and the median duration of current incarceration was 12 months (range 0.25–360; SD ± 30.86). The median number of cellmates was 12 incarcerated persons (range 1–60; SD ± 12.61).

Most participants (63.7%) reported irregular condom use. History of having engaged in homosexual intercourse was declared by 6.7% of the participants and homosexual preference by 3.1%. Thirty-five percent of the participants had five or more sexual partners in the previous five years. History of STI was reported by 10.2%. Part of the participants, 4.3% and 35.0%, reported ever having sexual intercourse with people who use drugs (PWUD) and with people who inject drugs (PWID), respectively.

Of 580 samples included in this study, total anti-HAV antibodies were detected in 511 of them (88.1%; 95% CI: 85.5–90.7). No sample had a positive reaction for IgM anti-HAV, suggesting that 88.1% of the participants had previously been exposed to HAV since none of the participants had received vaccination against hepatitis A. Prisons located in the cities of Campo Grande and Corumbá had higher anti-HAV prevalence than the other prisons enrolled in this study, 90.9% (95% CI: 87.6–94.1) and 98.3% (95% CI: 91.0–99.7), respectively. Among susceptible participants to HAV infection (negative serological test for total anti-HAV antibodies), one had positivity for the anti-HCV marker. All male participants who reported a history of homosexual intercourse were anti-HAV positive. Furthermore, all participants infected with HIV and 92.3% with positive syphilis status had positivity for anti-HAV. There was no statistical association between HAV infection positivity and anti-HIV (7/7), total anti-HBc (50/52), anti-HCV (11/12), and anti-*Treponema pallidum* positivity (48/52).

The HAV exposure prevalence increased with age, from 69.6% for participants 18–19 years of age to 72.0% for those 20–24 years, 88.3% for 25–29 years of age, 93.65% for 30–35 years, and 99.3% for prisoners > 35 years of age. This age-related trend in the prevalence of HAV exposure was statistically significant (p<0.001).

Table 1 presents the sociodemographic and behavioral characteristics associated with anti-HAV seropositivity. In univariate analysis, four variables were associated with HAV exposure: being imprisoned at Campo Grande (p = 0.01) or Corumbá (p = 0.01) cities, older age (p<0.01), low levels of education (p<0.01) and higher number of sexual partners in the previous 5 years (p<0.01). After multivariate logistic regression analysis, older age [odds ratio (OR) 6.58; 95% confidence interval (CI) 2.90–14.94], low level of education (OR 3.12; 95% CI 1.68–5.78 and OR 3.29; 95% CI 1.15–9.44) and being imprisoned in Corumbá city (OR 12.28; 95% CI 1.48–102.04) were independently associated with HAV exposure infection.

**Table 1:** Factors associated with HAV exposure infection among prisoners in Central Brazil (n = 580).

Variables	Anti-HAV IgG positive		OR (95% CI)	*p-*value	Adjusted OR (95% CI)	*p-*value
	N	(%)				
**Penitentiary city**						
Dourados	72/89	80.9	1		1	
Três Lagoas	51/63	81.0	1.00 (0.44–2.28)	0.99	0.94 (0.36–2.45)	0.90
Ponta Porã	52/63	81.5	1.12 (0.48–2.58)	0.80	0.82 (0.31–3.00)	0.69
Campo Grande	278/306	90.9	2.34 (1.22–4.52)	0.01	1.63 (0.75–3.58)	0.22
Corumbá	58/59	98.3	13.69 (1.77–105.98)	0.01	12.28 (1.48–102.04)	0.02
**Age (years)**						
<30	242/302	80.1	1		1	
≥30	269/278	96.8	7.41 (3.60–15.25)	<0.01	6.58 (2.90–14.94)	<0.01
**Education (years)** ^**a**^ ^,^ ^**b**^						
> 9	122/161	75.8	1		1	
5–9	277/302	91.7	3.54 (2.05–6.11)	<0.01	3.12 (1.68–5.78)	<0.01
≤ 4	104/109	95.4	6.65 (2.53–17.49)	<0.01	3.29 (1.15–9.44)	0.03
**Number of incarcerated persons per cell** ^**a**^						
1–10	199/235	84.7	1		1	
11–20	185/210	88.1	1.34 (0.77–2.32)	0.30	1.47 (0.74–2.93)	0.26
>20	122/130	93.8	2.76 (1.24–6.13)	0.01	1.65 (0.60–4.54)	0.33
**Alcohol consumption** ^**a**^						
No	227/250	90.8	1		1	
Yes	275/319	86.2	0.63 (0.37–1.08)	0.09	0.80 (0.42–1.52)	0.49
**History of transfusion** ^**a**^						
No	425/488	87.1	1		1	
Yes	77/83	92.8	1.90 (0.80–4.55)	0.15	1.07 (0.41–2.78)	0.89
**Sex with non-injecting drug user** ^**a**^						
No	336/374	89.8	1		1	
Yes	172/203	84.7	0.63 (0.38–1.04)	0.07	0.57 (0.31–1.06)	0.08
**Number of sexual partners in the last 5 years** ^**a**^						
≤ 5	370/407	90.9	1		1	
> 5	141/173	81.5	0.44 (0.26–0.73)	<00.1	0.739 (0.41–1.34)	0.32
**Previously had homosexual contact** ^**a**^						
No	452/518	87.3	1		1	
Yes	38/39	97.4	5.55 (0.75–41.09)	0.09	3.65 (0.44–29.94)	0.23
**Illicit drugs** ^**a**^						
No	258/285	90.5	1		1	
Yes	248/289	85.8	0.63 (0.38–1.06)	0.08	1.06 (0.56–2.01)	0.85
**Total anti-HBc**						
Negative	461/528	87.3	1		1	
Positive	50/52	96.2	3.63 (0.86–15.28)	0.08	1.67 (0.35–7.91)	0.52

95% CI: 95% confidence interval

OR: Odds ratio

^a^The total represents the number of individuals who answered the question

^b^All illiterate participants were positive for anti-HAV (6/6) and were included among participants with less than or equal to 4 years of study.

## Discussion

Epidemiological investigations about HAV infection are essential instruments to evaluate the public health strategies to prevent, control, and treat this injury that affects the prison population. Furthermore, this is the first nationwide investigation of HAV exposure among prison inmates. The prevalence rate of HAV exposure found among incarcerated persons in this study (88.1%; 95% CI: 85.5–90.7) was higher than the overall rate found in a population-based study conducted in capital cities of the North, Northeast, and Midwest in the general population of Brazil (68.8%; 95% CI: 64.8–72.5) (P<0.001). However, if the same age group is considered (≥ 20 years), both prevalence rates become similar [10]. Another national study in the Midwest region showed an anti-HAV prevalence rate of 56.0% in a group aged 10 to 19 years [11].

A similar high prevalence of HAV exposure was also found in studies conducted in Italy (86.4%) and Switzerland (95.7%). Although these countries have low endemicity for HAV infection, these prevalence rates were found in foreign prisoners [3, 6].

This high prevalence rate of HAV exposure has been associated with some risk factors observed among prisoners both before (social vulnerability) and during incarceration (overcrowding, poor hygiene, and sanitation) [7, 12].

Exposure infection to HAV was strongly associated with older age and low education levels among the participants. This fact is probably caused by environmental improvements both before (social vulnerability such as lack of hygienic-sanitary conditions and access to safe water) and during incarceration (overcrowding, poor hygiene, and sanitation), shifting the acquisition of HAV infection to later in life [1].

Sexually transmitted infections are considered risk factors for HAV infection, such as syphilis and HIV. According to published studies, syphilis lesions facilitate the entry of other infections, and HIV infection increases the period of HAV viremia, leading to more efficient infection transmission [13, 14]. Even though both infections could share a common route of transmission, there was no statistical association in the present study. Furthermore, previous studies also pointed out that acquiring HAV at higher age and preexisting chronic liver disease (e.g., caused by hepatitis B or C virus) are important risk factors for HAV infection morbidity and mortality [3].

About 70% of the study participants had no more than primary education, probably caused by poor access to school. Studies indicate that HAV infection occurs more frequently in populations with low socioeconomic status [1]. Socioeconomic factors, including household income and education level, are better indicators of HAV seroprevalence than access to drinking water, according to some studies [1, 10].

The prevalence rate of HAV exposure in the participants from Corumbá (98.3%; 95% CI: 91.0–99.7) and Campo Grande (90.9%; 95% CI: 87.6–94.1) was similar, considering the overlap of Confidence Interval. This fact is not observed among Corumbá and the other cities. Therefore, in these other cities, the HAV exposure prevalence among adult individuals is similar to the Brazilian prevalence [age group of 20–24 years (82.2%; 95% CI: 67.9–92.0)] [10].

The environment of closed penal institutions and the risky behavior pattern of prisoners may lead to an increasing number and severity of HAV infection cases [2, 3, 12]. In the present study, imprisonment in Corumbá city was associated with HAV exposure. Sanitation data about the five cities involved in this study revealed that the population of Corumbá city has the poorest access to sanitation of all cities where the study was conducted. Just 19.3% of Corumbá’s city population has appropriate access to sanitary sewage compared to 51.5% of the total population of the Midwest region [15].

Thus, it is suggested that the sanitary conditions of the city could interfere with the prison environment. Conditions of overcrowding, lack of adequate sanitation, and poor hygiene are associated with HAV prevalence [1–3].

The main limitation of this study is related to the veracity of participants’ responses to risk behaviors due to social stigma and discrimination. It may reduce the statistical power of factors associated with HAV exposure. The study did not include some factors potentially associated with HAV infection, such as travel to endemic areas, migration, and household income. Only a part of a larger sample of incarcerated persons was used in this study and might not reflect the real scenario of the entire inmate group. Nevertheless, the sampling used was enough to prevent underestimated results according to the sample size calculation performed.

## Conclusion

The prevalence rate of HAV exposure found among incarcerated persons was classified as high but similar to the prevalence among the general Brazilian population of the same age group. Old age, lower level of education, and being imprisoned in Corumbá city were factors independently associated with HAV exposure. Thus, vaccination should be considered for susceptible prisoners to prevent new HAV infections, at least among those who have older age, preexisting hepatic chronic disease, and risk factors for acquiring this infection (UDI and MSM) [7]. Therefore, these strategies may avoid the economic burden of the disease and its potential dissemination inside the institutions and to the general population.
